# A Simple Protocol for Sample Preparation for Scanning Electron Microscopic Imaging Allows Quick Screening of Nanomaterials Adhering to Cell Surface

**DOI:** 10.3390/ijms24010430

**Published:** 2022-12-27

**Authors:** Anca Emanuela Minuti, Luminita Labusca, Dumitru-Daniel Herea, George Stoian, Horia Chiriac, Nicoleta Lupu

**Affiliations:** 1 National Institute of Research and Development for Technical Physics, 700050 Iasi, Romania; 2 Faculty of Physics, Alexandru Ioan Cuza University, 700506 Iasi, Romania

**Keywords:** SEM imaging, cell samples, magnetic nanoparticles, nanowires

## Abstract

Preparing biological specimens for scanning electron microscopy (SEM) can be difficult to implement, as it requires specialized equipment and materials as well as the training of dedicated personnel. Moreover, the procedure often results in damage to the samples to be analyzed. This work presents a protocol for the preparation of biological samples to evaluate the adherence of nanomaterials on the cell surface using SEM. To this end, we used silicon wafers as a substrate to grow cells and replaced difficult steps such as the critical point drying of the samples in order to make the method quicker and easier to perform. The new protocol was tested using two different types of cells, i.e., human osteosarcoma cells and adipose-derived mesenchymal stem cells, and it proved that it can grossly preserve cell integrity in order to be used to estimate nanomaterials’ interaction with cell surfaces.

## 1. Introduction

When assessing the interaction between nanomaterials and cells, an important step is to visualize the effects induced by the material to the cell membrane. In order to evaluate the results of the interaction process, SEM is typically used. Commonly, an important step during the preparation of biological cell samples for SEM is represented by critical point drying, which involves the replacement of the alcohol used for dehydration with an inert gas in order to preserve the cell morphology [1]. This conventional drying method is not only hard to accomplish, but it can lead to sample destruction if specific parameters are not met [2]. Alternatively, another method used to preserve cell morphology is replacing the ethanol necessary for dehydration with hexamethyldisilazane, a highly toxic and flammable compound that that can induce severe burns and respiratory problems if not properly managed [3]. On the other hand, when assessing nanomaterials adhered to the cell membrane, maintaining the shape of the cell is not necessarily important, so a little cell deflation does not affect the intended purpose of the evaluation. With this in mind, we propose replacing critical point drying (CPD) with the air drying of samples in a biological safety hood after dehydrating them.

When using magnetic nanoparticles or nanowires for cancer targeting, an important step is to assess the presence of the magnetic material in relation with the cells we are targeting. One method of cancer treatment is magnetic hyperthermia, which involves the heating of magnetic particles through the magnetic fields applied, which in turn heat the cells until inducing their deaths [4]. For this application to work, we need to ensure the presence of magnetic particles on the cell surface, as well as their distribution on the membrane. As a consequence, we need to obtain reliable images of the cell–nanoparticle interaction to assess if a suitable quantity adheres on their surface.

Here, we describe a simple and more cost-efficient method to prepare biological samples for SEM imaging which preserves cell integrity and can be used to describe nanomaterials’ interaction with cell surface.

## 2. Results

### 2.1. Images of Cells Obtained Using an Inverted Microscope

**Figure 1:**
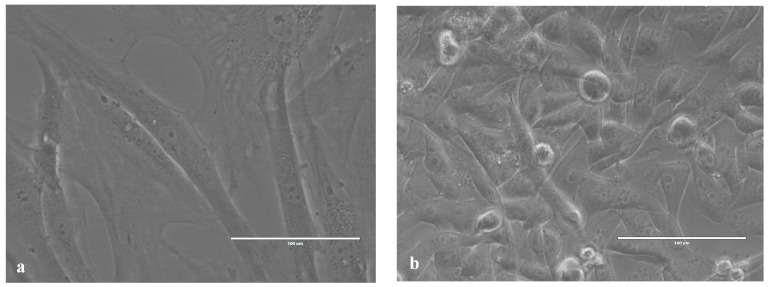
(**a**) Adipose-derived mesenchymal stem cells; (**b**) human osteosarcoma cells.

Before fixation for SEM imaging, the cells were routinely checked using an inverted microscope. Figure 1 contains two images of HOS and ADSC cell cultures.

### 2.2. Images of Cells without Nanomaterials Obtained Using the Proposed Protocol

**Figure 2:**
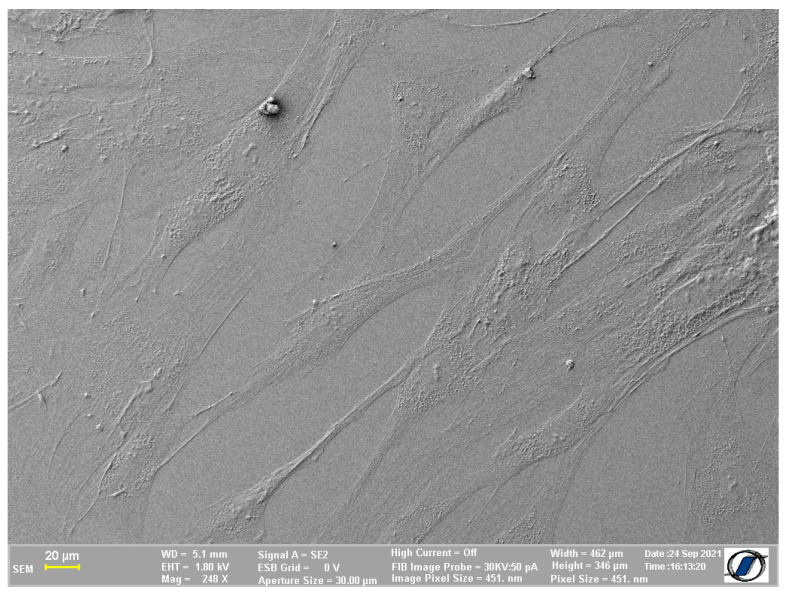
Adipose-derived mesenchymal stem cells—air dried.

When using the protocol we propose, one of possible pitfalls is cell deflation induced by the lack of inert gas (which is commonly used in traditional protocols [2,3]) to replace the liquid eliminated by drying the sample. However, for membrane imaging purposes, the protocol we propose allows for the detailed description of cell–nanoparticle interaction, even in a situation in which larger cells are used. This proves that the method is convenient to use in order to obtain proper images of the cell membrane without losing essential details. Figure 2 presents SEM imaging of adipose-derived mesenchymal cells (ADSCs) normally characterized by their spindle shape when adherent on a flat cell culture surface. Although they appear to be a little flatter than they usually are in 2D cultures, the degree of deflation is lower than previously expected and does not affect the appearance of the cells.

**Figure 3:**
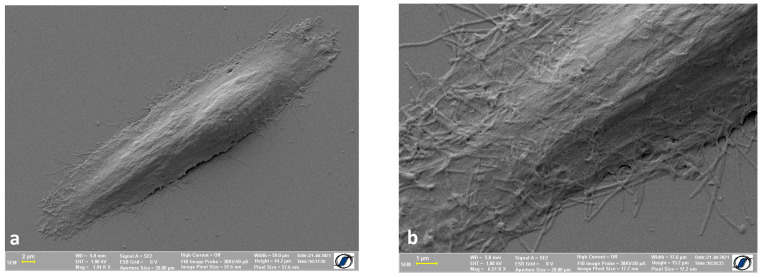
Human osteosarcoma cells—air dried: (**a**) the entire cell; (**b**) close-up of the cell membrane with all the details of it.

We also tested the protocol using a smaller cell type, a human osteosarcoma cell line (HOS). It is possible that the HOS cell body, being smaller and rounder (as seen in optical microscopy), undergoes deflation to a lesser extent compared to ADSC, which requires further confirmation. In Figure 3a, we are able to display an entire osteosarcoma cell, showing many of the cell protrusions and other details of the cell surface, further highlighted in Figure 3b, which shows clearer features of the cell surface which will help us evaluate the process of nanomaterials adhesion to the cell surface.

### 2.3. Images of Control Cells (without Nanomaterials) Obtained Using CPD

**Figure 4:**
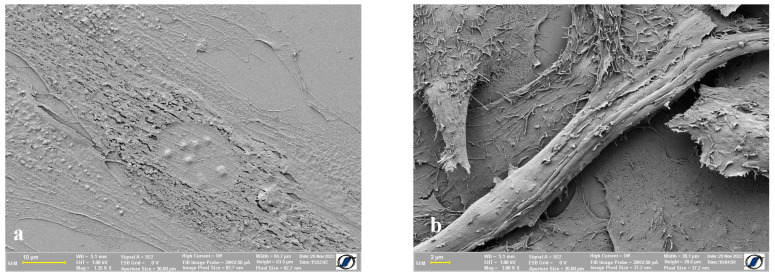
Cells dried using CPD; (**a**) adipose-derived mesenchymal stem cells; (**b**) human osteosarcoma cells.

To better compare the differences between air drying the samples and using CPD to do so, we prepared specimens with HOS and ADSCs on which we used critical point drying. For the samples we were able to obtain, ADSCs (Figure 4a) proved to be harder to preserve, as we were not able to avert the apparition of cracks on the cell membrane. In terms of the HOS cells, the images we were able to obtain appear to have more defined details on the cell surface, as opposed to the images in Figure 3.

We also measured the length and width of the cells (ADSC and HOS). For ADSCs, the average length was found to be 196 µm, and the width was ~28 µm when dried in the method we proposed, and they were 201 µm in length and ~25 µm wide when dried using critical point drying. As for HOS, when dried in the biological safety hood, the average length was 43.18 µm and 13.35 µm wide, while the same cells were 44.45 µm long and 13 µm wide when sample processing included critical point drying. The cells were measured to see if a change in the type of dehydration process used to process the samples induced any change to the size of the cells.

### 2.4. Images of Cells with Nanomaterials on the Cell Surface

**Figure 5:**
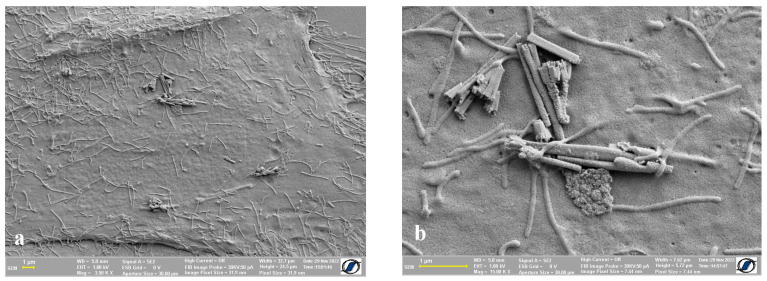
Human osteosarcoma cells with Fe-Co nanowires: (**a**) an agglomeration of Fe-Co nanowires on the cell surface; (**b**) close-up of the nanowires adherent on the cell surface.

**Figure 6:**
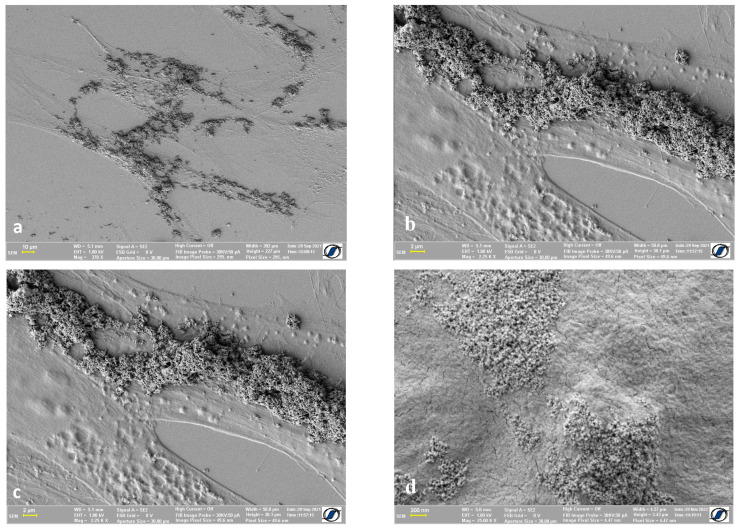
Adipose-derived mesenchymal stem cells with Fe-Cr-Nb-B magnetic nanoparticles: (**a**) clusters of magnetic particles on the cell membrane; (**b**–**d**) close-up of the magnetic particles adherent on the cell surface.

Regarding nanomaterials adherent on the cell surface, it is worth mentioning that the multiple washing steps required to accomplish the protocol can be indirect proof that the nanowires and other magnetic nanomaterials, which can be seen in the images from Figure 5 and Figure 6, were very well attached to the cell membrane. In Figure 5, we show human osteosarcoma cells with an aggregation of nanowires on the cell surface. The nanowires were too large and not dispersed enough to enable the internalization inside the cells, but they were adequately attached to the cell membrane so that they could not be washed away.

Figure 6 provides a clear image of the way Fe-Cr-Nb-B magnetic nanoparticles (MNPs) attach to the cell surface of adipose-derived mesenchymal stem cells. The size of the magnetic particles ranged between 10 and 200 nm, small enough to be internalized. The internalization process is time-dependent, usually taking between 2 and 24 h, as previously reported [5]; therefore, depending on the timing of cell preparation for SEM, a portion of MNPs could be visualized on the cell surface.

**Figure 7:**
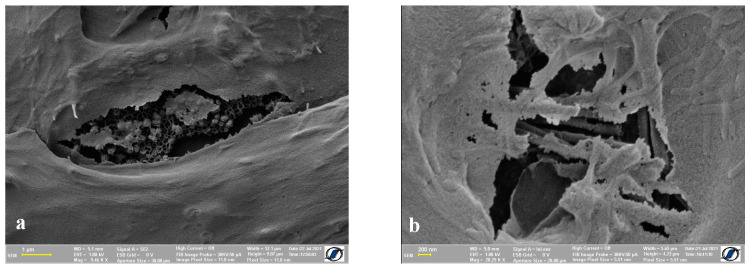
SEM imaging with (**a**) ADSC and (**b**) HOS exhibiting membrane disruption.

One of the downsides of this method compared to the use of critical point drying is the possibility of the appearance of cracks on the surface of the cells. In Figure 7, we can see a few examples of the anomalies displayed by the resulting samples. The cell membrane of both types of cells we used showed small, isolated cracks, but their appearance did not affect the purpose of this protocol, which is the evaluation of adherent materials on a cell surface. However, the method can be tailored to minimize these drawbacks by shortening the time needed to dry the samples and by careful manipulation of the samples.

## 3. Discussion

The interaction between nanomaterials such as magnetic nanoparticles and nanowires is an intensely researched subject due to their many biotechnology applications. In particular, magnetic nanoparticles are widely studied in relation to applications such as use as contrast agents for magnetic resonance imaging [6], drug delivery [7], and regenerative medicine [8]. For this reason, it is important to have a reliable and efficient method to examine the interaction between nanomaterials and cells in high resolution. SEM imaging can provide reliable visualization of nanomaterial interaction with cell surfaces on the condition the samples are properly processed. For applications in which the magnetic nanomaterials need to at least be on the cell surface, namely tumor targeting [9], it is important to not only quantify the amount of nanomaterial attached on cell membranes, but also to ensure its integrity is not affected by their presence. Furthermore, SEM imaging provides clearer images of specimens than light microscopy can produce, as the resolution and magnification of the former is higher than the latter.

Critical point drying for the preparation of biological samples for SEM imaging is considered the lengthiest and hardest to implement part out of the earlier protocols [10]. CPD is a method of drying samples by replacing water with a transitional liquid, usually ethanol, followed by replacing it with a liquid gas, such as CO_2_ [11]. For this step, samples are usually inserted in the CPD device and flooded with liquid gas several times at certain pressures and temperatures, a procedure that can affect the integrity of the membrane just as much as not replacing the liquids from the cell when air drying. Other reports exist regarding circumventing CPD and occasionally even the use of heavy metal treatment for different purposes, such as observing cell surface structure and cell to cell interaction [12], the visualization of gene delivery vectors within HeLa cells [13], or cell surface visualization [14]. All the above-mentioned reports involve the air drying and successive organic solvent desiccation of cells. However, we report—to our knowledge, for the first time—a method involving culturing cells directly on silicon wafers routinely used for SEM imaging for the specific purpose of visualizing cell surface interaction with various nanomaterials.

Magnetic nanoparticles and nanowires were clearly observed in the images obtained using the method presented above. While the cell surface was minimally affected by the drying process applied, the resulting images clearly depicted cell surface interaction with the nanomaterials. This observation was systematic in all the samples, confirming the fact that the method we describe can be used for this purpose. Furthermore, the samples in which slight cell deflation occurred could still preserve cell morphology and succeeded in visualizing cell surface–nanomaterial interaction for the cell types used in this study.

Most of the images obtained from smaller cells such as osteosarcoma cells highlighted the entire cell membrane without showing apparent breakage, while larger cells such as ADSCs showed small cracks in the cell membrane, depending on the height of the cell. Although the method has a few already-mentioned possible pitfalls, the advantages of the method far surpasses them, as the method is not only easy and cost effective to replicate but also leads to valid results. Furthermore, the cell preparation protocol we propose does not require special training to use to the equipment, being accessible to a larger number of working conditions.

## 4. Materials and Methods

### 4.1. Cell Growth and Nanomaterial Adherence on the Cell Surface

We used silicon wafers as substrate for cell growth. Before seeding cells on such substrates, the silicon wafers were washed using deionized water and sterilized using an autoclave. They were then placed into Petri dishes and washed once with complete cell culture media (CCM) to prepare the surface for the adherence of cells. The chosen cell types were trypsinized, counted, and the required number was added to the Petri dishes (for the 35 mm Petri dish, we incubated 3 × 10^5^ cells in 2 mL of CCM). The cells were grown to 80% confluency and the nanomaterials, such as magnetic nanoparticles and nanowires, were dispersed in fresh cell culture media and added onto the cell culture. The cells were co-incubated with the nanomaterial for 24 h depending on the purpose of the study.

### 4.2. Cell Fixation and Preparation for SEM Imaging

After the period of co-incubation, the cell culture media were taken out, and the samples were washed with PBS to eliminate the excess of nanomaterial that was not adhered on the cell membrane as well as the residual CCM from the sample. For the fixation of the cell samples, we added a solution of 2.5% glutaraldehyde in phosphate buffer for 2 h at room temperature. Afterwards, the cells were washed with phosphate buffer three times for 15 min each wash. The cells were then post-fixed in 1% osmium tetroxide (Electron Microscopy Sciences, Hatfield, PA, USA) in phosphate buffer for 1 h in the dark, followed by washing with deionized water three times for 15 min each. The washing process was followed by serial dehydration using ethanol at 10%, 25%, 40%, 50%, 70%, 90%, and 100% for 5 min each. Subsequent to the dehydration process, the silicon wafers were air dried for 3–10 h in the biological safety hood without the lid on, and then, they were vacuum dried to prepare for film coating with gold. The 5 nm thick gold film was used to prevent surface charge buildup of the sample without losing the details on the cell membrane. For the specimens we dried using CPD, after the last step of dehydration, the samples were moved in the CPD equipment chambers, designed for biological samples, a process that led to the replacement of alcohol with CO_2_ gas. Afterwards, these samples were also coated with a gold film, as described above.

Lastly, the biological samples were imaged using a scanning electron microscope (FIB/FE-SEM CrossBeam Carl Zeiss NEON 40 EsB). The imaging mode used was Secondary Electron Detector, working distance 5.1 mm, 1.8 kv voltage, and aperture diameter between 20 and 30 µm.

In this protocol, we tested the method using human osteosarcoma cells (HOS; MG-63) and adipose-derived mesenchymal cells (ACSs). To evaluate the nanoparticles’ adherence, we used magnetic nanoparticles obtained via ball milling of Fe-Cr-Nb-B superferromagnetic ribbons dispersed in calcium gluconate to prepare a ferrofluid which was later dispersed in complete cell culture media [15]. We also tested the adherence of Fe_64_._5_Co_35_._5_ nanowires with lengths of approximatively 4 microns which were obtained via electrolytic deposition in alumina membranes with a pore diameter of 200 nm [16].

## 5. Conclusions

Using an updated version of air drying, glutaraldehyde-based stabilization, and progressive organic solvent desiccation, as well as an adapted method of cell culture on SEM silicon wafers, we were able to obtain realistically preserved biological cell samples, both with and without nanomaterials adhered to the cell membrane surface. The obtained images displayed details of the cell surface, even with the added layer of gold film. Nanomaterials such as magnetic nanoparticles and magnetic nanowires were easily traceable on two types of cell surfaces (human normal primary cells and a cancer cell line).

## Data Availability

The data presented in this study are available on request from the corresponding author.
